# Linoleic Acid Induced Acute Hepatitis: A Case Report and Review of the Literature

**DOI:** 10.1155/2015/807354

**Published:** 2015-07-09

**Authors:** Mohammad Bilal, Yogesh Patel, Micheal Burkitt, Michael Babich

**Affiliations:** Department of Gastroenterology & Hepatology, Allegheny General Hospital, 320 East North Avenue, Pittsburgh, PA 15212, USA

## Abstract

Several dietary supplements used for weight loss have been reported to cause hepatotoxicity. Conjugated Linoleic Acid (CLA) is a dietary supplement that has been shown to cause reduction in body fat mass. Here, we present the first case of CLA induced acute hepatitis in the United States and only the third case in the worldwide literature along with a brief review of the literature.

## 1. Introduction

Several dietary supplements used for weight loss have been reported to cause hepatotoxicity [1]. Conjugated Linoleic Acid (CLA) is a dietary supplement that has been shown to cause reduction in body fat mass [2, 3]. Here, we present the first case of CLA induced acute hepatitis in the United States along with a brief review of literature.

## 2. Case Report

A 26-year-old female with a past history of obesity and sickle cell trait presented to the hospital with complaints of right upper quadrant abdominal pain and vomiting for one day. The patient was not using any prescription medication. The patient had intentionally lost 50 pounds of weight within the last six months. The patient was going to the gym regularly and was following a strict diet. One week prior to presentation to the hospital, the patient started using CLA supplements to help in weight loss. Physical exam was unremarkable except for mild right upper quadrant abdominal tenderness. There was no jaundice, hepatomegaly, or stigmata of chronic liver disease seen.

**Figure 1:**
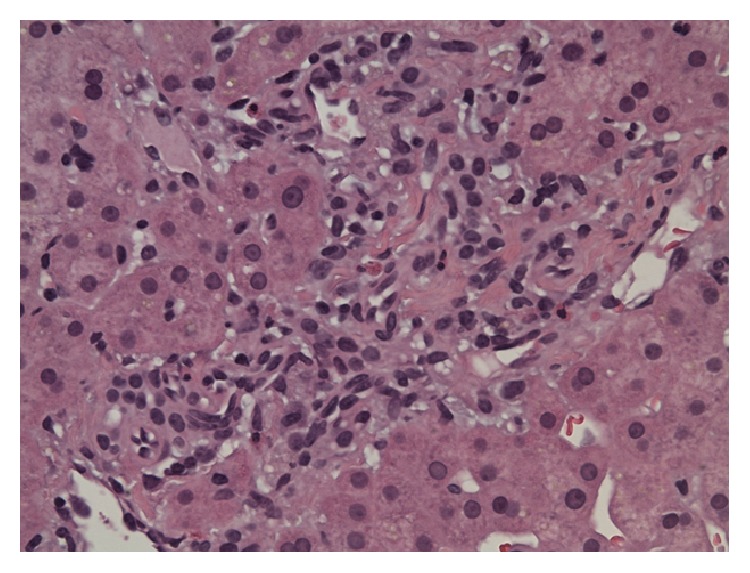
Representative portal area with few eosinophils but no evidence of interface hepatitis or bile duct injury.

**Figure 2:**
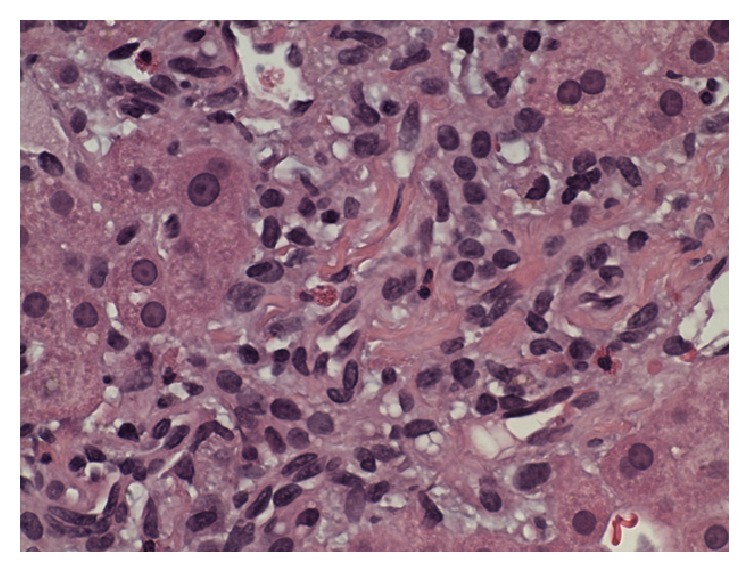
Portal tract with few eosinophils.

Laboratory investigations revealed the following: total bilirubin: 2.0 mg/dL, aspartate aminotransferase (AST): 1519 U/L, alanine aminotransferase (ALT): 1078 U/L, alkaline phosphatase (ALP): 111 U/L, gamma-glutamyl transferase: 368 U/L, prothrombin time: 15.1 seconds, and international normalized ratio: 1.2. White blood cell count was normal. A right upper quadrant ultrasound revealed a normal acoustic texture and echogenicity in the liver parenchyma and no liver mass was seen. It also showed cholelithiasis but no evidence of gall bladder wall thickening or pericholecystic fluid. The common bile duct was 0.4 cm in diameter. There was no intrahepatic biliary dilatation seen and no ascites was present. Serology results for hepatitis A, hepatitis B, and hepatitis C were negative. Results were also negative for anti-mitochondrial, anti-nuclear, and anti-smooth muscle antibodies. Serum alpha fetoprotein, alpha-1-antitrypsin, ceruloplasmin, and ferritin levels were in the normal range. Urine drug screen was negative for benzodiazepines, barbiturates, cannabinoids, opioids, cocaine, amphetamine, methamphetamine, and PCP. Acetaminophen level was undetectable. Finally, a liver biopsy was done which revealed increase in sinusoidal macrophages and lymphocytes in the lobules. Most portal areas showed small number of eosinophils and macrophages (Figures 1 and 2). Trichrome stain was negative for fibrosis. Iron staining was also negative. No interface hepatitis or bile duct injury was seen. Based on the clinical, laboratory, and pathological data, a diagnosis of acute hepatitis was made. CLA therapy was discontinued. The patient was managed conservatively with intravenous fluids and antiemetics. The patient's nausea, vomiting, and abdominal pain resolved the next day. The patient started tolerating a regular diet and did not have any recurrence of abdominal pain or nausea. On day three of hospitalization, the patient's aminotransferases stabilized and the patient was discharged to followup in one week. One week later, the patient's aminotransferases had fallen considerably and the patient had no recurrence of symptoms (Table 1).

## 3. Discussion 

The use of dietary supplements is becoming increasingly popular in the United States [4]. Some studies have reported that almost half of the United States adult population uses herbal or dietary supplements [1]. A number of dietary supplements have been reported to cause acute hepatitis [1, 5]. The spectrum of hepatotoxicity has been variable, ranging from mild hepatitis to acute hepatic failure requiring liver transplantation.

CLA is a polyunsaturated omega-6 fatty acid. It is sometimes used by patients to aid in weight loss. There have been some studies which have reported reduction in body fat mass [2, 3, 6]. The two bioactive isomers* cis-9, trans-11 and trans-10, cis-12 CLA* have been shown to stimulate immune responses and improve insulin sensitivity [3, 7]. CLA is also reported to beneficially modify lipid metabolism [8]. Fatty acid analysis in one study revealed that the cis-9, trans-11 CLA isomer was incorporated into total plasma lipids following supplementation with both isomeric blends of CLA. The same study demonstrated improvement in plasma triacylglycerol-rich lipoprotein and VLDL cholesterol metabolism, leading to the conclusion that CLA has some cardioprotective roles in humans [8].

To the best of our knowledge, this is the first case of CLA induced acute hepatitis in the United States and the third case in the worldwide literature [9, 10]. The biochemical presentation of this patient and the prior two patients has been that of primarily hepatocellular damage with elevations in AST and ALT. One previously reported patient had complete resolution of symptoms and aminotransferases after discontinuation of CLA, while the other patient had to undergo emergent liver transplantation. All three patients had no other risk factors for acute hepatitis and had started using CLA within the last 30 days prior to presentation. In the two cases previously reported, serum bilirubin was significantly elevated. In the patient requiring liver transplantation, serum bilirubin was 26 mg/dL as compared to 12.9 mg/dL in the other patient [9, 10]. The serum bilirubin was 2.0 mg/dL in our patient. This may point towards correlation in the severity of disease with serum bilirubin levels rather than the aminotransferases. However, there is not enough data to support this conclusion.

The Drug-Induced Liver Injury Network (DILIN) reported the use of herbal and dietary supplements as the second most common cause of drug induced liver injury in the United States. In a prospective multicenter study conducted by DILIN, 15.5% of patients who had drug induced liver injury were found to be using herbal or dietary supplements. 69% of the patients who had liver injury secondary to herbal and dietary supplements had to be hospitalized, while 8.2% of the patients had to undergo emergent liver transplantation [1].

This case demonstrates that herbal and dietary supplements are not always safe. This also stresses the need for physicians to counsel patients about the use of dietary supplements and to be aware of CLA use as a potential cause of hepatotoxicity.

## Figures and Tables

**Table 1:** Trend of aminotransferases.

	Day 1	Day 2	Day 3	Day 7
Total bilirubin (mg/dL)	2.0	1.1	1.0	0.7
ALP (U/L)	111	142	144	92
ALT (U/L)	1078	1223	1247	204
AST (U/L)	1519	1300	1047	36
